# Scaled-Up Mobile Phone Intervention for HIV Care and Treatment: Protocol for a Facility Randomized Controlled Trial

**DOI:** 10.2196/resprot.3659

**Published:** 2015-01-23

**Authors:** Kelly L L'Engle, Kimberly Green, Stacey M Succop, Amos Laar, Samuel Wambugu

**Affiliations:** ^1^FHI 360Social and Behavioral Health SciencesDurham, NCUnited States; ^2^FHI 360GhanaAccraGhana; ^3^FHI 360Scientific AffairsDurham, NCUnited States; ^4^School of Public HealthUniversity of GhanaAccraGhana

**Keywords:** implementation science, mobile phones, mHealth, HIV, AIDS, HIV care and treatment, cluster-RCT, Ghana

## Abstract

**Background:**

Adherence to prevention, care, and treatment recommendations among people living with HIV (PLHIV) is a critical challenge. Yet good clinical outcomes depend on consistent, high adherence to antiretroviral therapy (ART) regimens. Mobile phones offer a promising means to improve patient adherence and health outcomes. However, limited information exists on the impact that mobile phones for health (mHealth) programs have on ART adherence or the behavior change processes through which such interventions may improve patient health, particularly among ongoing clients enrolled in large public sector HIV service delivery programs and key populations such as men who have sex with men (MSM) and female sex workers (FSW).

**Objective:**

Our aim is to evaluate an mHealth intervention where text message reminders are used as supportive tools for health providers and as motivators and reminders for ART clients to adhere to treatment and remain linked to care in Ghana. Using an implementation science framework, we seek to: (1) evaluate mHealth intervention effects on patient adherence and health outcomes, (2) examine the delivery of the mHealth intervention for improving HIV care and treatment, and (3) assess the cost-effectiveness of the mHealth intervention.

**Methods:**

The 36-month study will use a facility cluster randomized controlled design (intervention vs standard of care) for evaluating the impact of mHealth on HIV care and treatment. Specifically, we will look at ART adherence, HIV viral load, retention in care, and condom use at 6 and 12-month follow-up. In addition, participant adoption and satisfaction with the program will be measured. This robust methodology will be complemented by qualitative interviews to obtain feedback on the motivational qualities of the program and benefits and challenges of delivery, especially for key populations. Cost-effectiveness will be assessed using incremental cost-effectiveness ratios, with health effects expressed in terms of viral load suppression and costs of resources used for the intervention.

**Results:**

This study and protocol was fully funded, but it was terminated prior to review from ethics boards and study implementation.

**Conclusions:**

This cluster-RCT would have provided insights into the health effects, motivational qualities, and cost-effectiveness of mHealth interventions for PLHIV in public sector settings. We are seeking funding from alternate sources to implement the trial.

## Introduction

### Background

Adherence to prevention, care, and treatment recommendations among people living with HIV (PLHIV) is a critical challenge facing HIV service delivery programs. Myriad personal, social, and systems level barriers influence adherence among PLHIV on antiretroviral therapy (ART). Difficulty managing treatment and forgetting to take medications or attend clinic appointments are common reasons for poor adherence [1-4]. Lack of social support, negative perceptions, poor communication with providers, and stigma and discrimination also may limit adherence [2,3]. In resource-poor settings, adherence challenges are amplified by structural and economic constraints such as residential dispersion that requires long travel times and has costs associated with travel and wage loss [3,4]. Key populations are disproportionately less likely to access and remain in care and treatment compared to the general population of PLHIV [5]. Good clinical outcomes depend on high adherence; consistent adherence to ART regimens is associated with reduced HIV RNA (viral load) levels, decreased transmission, higher CD4 cell counts, lower health care costs, and overall improved quality of life [6-9].

Mobile phones offer a promising means to improve patient adherence and health outcomes because they are private, portable, increasingly affordable, and nearly ubiquitous (88% use in Ghana [10]). Mobile phone interventions provide a means to address several of the key barriers to good adherence by providing reminders for care and a direct connection to health providers and facilities [11,12]. Research conducted in East Africa has shown that texting (short message service, SMS) reminders to ART clients’ mobile phones improved adherence and health [13-15]. Due to favorable findings from mobile phones for health (mHealth) interventions, limited proven adherence and treatment support interventions [3,16,17], and the need for a combination of interventions to address the complexities of adherence, mHealth programs are rapidly being scaled up globally [18,19].

### Justification for Study

Implementation research is needed to keep pace with mHealth program scale-up to provide guidance for optimal delivery and effectiveness of mHealth programs [3,18,20-22]. Although mobile phone reminders have been shown to improve medication adherence among new ART clients in a few health facilities, a trial conducted in one health facility in Cameroon with continuing ART clients did not improve adherence [21]. We have limited information about mHealth program effects on continuing ART clients and subpopulations in large-scale, public-sector HIV care and treatment programs where the majority of PLHIV in Ghana seek HIV care. Since women and key populations eg, female sex workers (FSW), and men who have sex with men (MSM), continue to be disproportionally affected by HIV, it is critical to assess effectiveness among these groups [3,5]. Moreover, the need for adherence support may be greater among continuing ART clients as opposed to new ART clients, as adherence challenges grow over time [23,24]. The support provided by mHealth interventions may influence ART clients’ adherence as well as retention in HIV care and treatment and adherence to other prevention and care directives such as use of condoms. Furthermore, there is little evidence about the motivational behavior change processes through which mHealth interventions improve patient health [25]. It is imperative to understand when, why, and how interventions work to develop a sound understanding of effectiveness to inform program scale-up [25,26]. Lastly, decision-makers and program implementers need mHealth cost-effectiveness data to guide resource allocation [14,27,28].

### Study Objectives

The main study goal is to evaluate the impact, implementation, and cost-effectiveness of a mobile phone adherence support intervention delivered to patients on ART in large-scale public sector health services in Ghana, especially for women and key populations. The study has three objectives: (1) to evaluate mHealth intervention effects on patient adherence and health outcomes; (2) to examine the delivery of the mHealth intervention for improving HIV care and treatment; and (3) to assess the cost-effectiveness of the mHealth intervention.

## Methods

### Study Design

This study is a two-arm, facility randomized, open, controlled trial. Of the public-sector health facilities in Ghana that provide ART to PLHIV in conjunction with the National AIDS Control Program (NACP)/Ghana Health Services (GHS), 40 will be randomly and equally allocated to receive either the mHealth intervention or standard of care. Approximately 1600 PLHIV who have been on ART for at least 6 months and who own a mobile phone will be enrolled. Data collection will occur at baseline and 6 and 12 months post-enrollment. A subsample of women and intervention participants from key populations including MSM and FSW, and health care providers and managers at intervention facilities, will be randomly selected to complete qualitative interviews at follow-up.

### Facility Eligibility and Recruitment

The study will be implemented in health facilities in five regions of Ghana that have sufficient numbers of people on ART in public sector facilities and are accessible to study staff for data collection and monitoring. Specifically, eligible facilities must have at least 150 patients enrolled on ART, follow the national Ministry of Health guidelines for ART care and treatment [29], be a NACP/GHS facility, and they must not be extremely difficult to access due to very remote location or poor infrastructure for reaching the facility. To maximize facility homogeneity while balancing representativeness of clinics and participants, facilities meeting eligibility criteria will be stratified by geography (urban and rural) and number of ART patients receiving services. Facilities then will be recruited for study participation in proportion to their representation in the eligible facility population across strata until the target number of facilities is attained.

### Participants

#### Eligibility

To be in the study, participants must be between 18-49 years old, currently receiving ART at public sector facilities, enrolled in ART for a minimum of 6 months, and own a mobile phone. They also must live near the study facility and plan on residing near it for 12 months following study enrollment. Study volunteers who are currently participating in another HIV adherence study or who were involved in the mHealth pilot intervention will be excluded.

#### Recruitment

Potential participants initially will be informed about the study by implementing partners at support groups and at the study clinics. In addition, peer educators who work with PLHIV will tell MSM and FSW about the study. Facility staff and clinicians also may apprise patients about the study. Interested PLHIV will be encouraged to speak with study staff at the facilities during predetermined study recruitment times; these times will be arranged during medication refill days or other times when substantive numbers of potential participants are likely to be in study facilities. Screening and enrollment will take place for interested volunteers in a private location in each health facility, where eligible participants will be enrolled until the target sample size is attained. Participants from intervention facilities will be enrolled into the mHealth intervention at enrollment. It is estimated that recruitment will take approximately 6 months to complete.

#### Informed Consent

Participation will require written informed consent. Informed consent will be administered in English or Twi, the local language.

### Randomization

Once facilities are identified and agree to participate, they will be randomized within strata (urban/rural and number of ART patients) to the intervention or control group. Facility assignment will be revealed to field staff just prior to study initiation. Group assignment is needed before enrolling individuals and no blinding is possible. Eligible individual participants will receive the intervention or standard of care according to the assignment of the facility where they receive their HIV care and ART medication.

### Intervention: LifeLine

The mobile phone intervention, termed LifeLine, was developed and piloted as one component of the Unites States Agency for International Develop (USAID) Ghana project, Strengthening HIV/AIDS Partnerships with Evidence-based Results (SHARPER). SHARPER partners with 25 local non-governmental organizations (NGOs), the Ghana AIDS Commission, and the Ghana Health Service to increase healthy behaviors and access to HIV prevention and care services among MSM and FSW and their intimate partners, and PLHIV and their partners. LifeLine is a one-way text messaging service; participants receive but do not respond to the messages via mobile phone. A local technological partner will provide support for the fully automated LifeLine program, including enrollment of LifeLine participants from mobile phone numbers provided by study staff.

The LifeLine intervention sends daily text message ART reminders at no cost to PLHIV upon enrollment into the program. The messages were developed with input from both PLHIV and members of key populations. Many different messages have been developed and will be rotated approximately every 3 months. Daily messages refer to “medication” rather than drugs or ART, and data from pilot testing showed high acceptability, recall, and sharing of messages. Sample LifeLine messages include:

Taking my medicine every day makes me stay healthy to work and take care of myself and family.”

It is my life and I will make sure that nothing stops me from taking my medicine.”

Good morning! How are you today? Please remember your medication. Stay blessed!”

### Control

In the control condition, participants will receive usual care. This consists of the essential package of services specified by the Ministry of Health Guidelines for Antiretroviral Therapy in Ghana [29]. Services include ART adherence monitoring through self-report and pill counts, in-depth discussion of ART adherence at each treatment visit, regular CD4 testing, management of sexually transmitted infections (STIs), management of opportunistic infections, referrals and linkages within and outside the health system, and regular patient reports through the health information system. For patients who have been on ART for 6 months or more, regular visits are scheduled every 3 to 6 months unless there is an urgent health need, and CD4 testing is conducted twice per year.

### Sample Size

We calculated that a sample size of 40 clinics with 40 completed participants per clinic on average for a total of 1600 PLHIV is needed to detect a 15% improvement in adherence at 12 months, with 84% power and a (two-tailed) significance level of 0.05. These calculations assume that, at baseline, 65% of enrolled participants will be defined as having good ART adherence according to national guidelines (>90% adherent in Ghana). We have assumed approximately 20% participant attrition over the life of the study and have increased participant recruitment targets accordingly (N=2000). These calculations assume an intra-class correlation coefficient (ICC) of 0.05 due to the enrollment of participants and randomization by facility. The correlation (stability) in self-reported adherence over time that occurs within both clinics and subjects is assumed to be .50.

### Study Measures

#### Health Outcomes

Study health outcomes are ART adherence [23,30,31], viral load, retention in care [32,33], and condom use (Table 1). Routine data collection for clinical care related to ART adherence and health outcomes and tracking already occurs in the public sector NACP/GHS health facilities. These data are collected electronically by trained Strategic Information/ Monitoring & Evaluation staff employed by each facility and supervised by the NACP/GHS. This will be augmented with a supplemental data collection form administered to all participants at enrollment and 6 and 12-month follow-ups.

**Table 1:** Overview of measures.

Measures		Indicators	Data source	M0	M6	M12
**Health Outcomes**						
	ART adherence	Self-report in given time period; self-report via visual analog scale; pharmacy refills	Clinical care data; pharmacy records	X	X	X
	Viral suppression	Undetectable plasma HIV viral load (<400 copies/ml)	Laboratory testing	X		X
	Retention in care	Client tracking outcomes: stopped treatment, known to be dead, or lost to follow-up	Clinical care data		X	X
	Condom use	Consistency of use and use at last sex with different partners (main, casual)	Clinical care data	X	X	X
**Implementation Measures**						
	Health behaviors	Self-efficacy for taking ART; perceived social support for adherence; motivation for adherence; perceived quality and access to providers/facilities	Supplemental data collection	X	X	X
	mHealth intervention adoption	Satisfaction with intervention; message receipt and recall; privacy and confidentiality concerns; message relevance and trust; message sharing; actions taken on receiving messages; use of additional mobile phone services	Supplemental data collection; qualitative interviews		X^a^	X^a^
	Fidelity of intervention delivery	Messages sent on-time; messages received	Technology system logs; supplemental data collection		X^a^	X^a^
	Provider perspectives	Benefits and challenges to intervention implementation; intervention impact on PLHIV quality of care; integration with health system	Qualitative interviews			X^b^
**Costing Measures**						
	Costs of routine care	Training; additional staff time; travel for counselors; other	Intervention tracking tool; costing forms	X	X	X
	Costs of intervention	Initial software and hardware/server costs; maintenance for software and server; maintenance for tech support; monthly reporting; SMS costs; message development	Intervention tracking tool; costing forms	X	X	X

^a^Measures administered in intervention facilities only.

^b^Provider interviews will take place approximately nine months after intervention initiation in intervention facilities only.

#### Implementation Measures

This aspect of the study will use a theory-based evaluation approach to assess how and why the mHealth intervention works for PLHIV and key populations on ART [26]. The research will focus on how the mHealth intervention addresses behavioral, social, and cognitive change strategies. Study participants in both intervention and control groups will be asked about their motivation for good health and management of HIV disease in the supplemental data collection form. These health behavior constructs may influence the primary study outcomes, and they may mediate the impact of the mHealth intervention on these outcomes. In addition, to assess reasons for and barriers to mHealth intervention adoption, as well as consistency and timeliness of receipt of text messages, a few questions will be added to the supplemental data collection form for intervention facilities only. Intervention fidelity also will be assessed through review of system logs from the technology provider to check for on-time and same-time of day delivery of text messages to intervention participants.

To explore implementation issues that may be unique for women and/or key populations living with HIV, qualitative in-depth interviews will further assess confidentiality and privacy concerns, message relevance and trust, and other program delivery and adoption issues, in addition to assessing opinions about the motivational aspects of the intervention. Interviews also will include questions about the social support function of mobile phones, probing on how the intervention may increase perceptions of social support from health care providers and PLHIV, as well as how social support may be increased by sharing of messages with family and friends. Finally, interviews will include questions about how participants’ use of the mHealth intervention may have affected their use of mobile phones for other information and services, particularly for women and key populations. A minimum of three interviews per intervention facility will be conducted.

The health care provider qualitative interviews will focus on benefits and challenges to implementing mHealth interventions in health facilities and with clients, as well as to integrating mHealth components into larger health systems in general. One health care provider and one manager will be interviewed in each facility.

#### Costing Measures

Costs will be assessed for implementing the standard of care in NACP/GHS facilities, as well as costs for implementing the LifeLine mHealth intervention. Information from NACP/GHS facility and SHARPER program records will be extracted to account for retrospective and prospective costs, detailed using an Intervention Tracking Tool (ITT) and accompanying prospective costing forms.

### Analysis

#### Primary Analyses

Generalized estimating equations (GEE) will be applied to compare intervention and control arms on the key 6 and 12-month health outcomes. In order to adjust for variance in the outcomes at the facility-level, compound symmetric working correlation matrices will be used in all models. In order to maximize statistical power, all models will control for baseline measures of the outcomes. In addition, all models will include sex, stratification variables, and time on ART, as well as other covariates identified from the literature and to be specified in the final statistical analysis plan. Hypotheses will be tested at the 5% significance level for two-sided comparisons.

#### Mediation Analyses

In addition, we plan to explore the mediating effects of health behavior constructs such as self-efficacy for taking ART and using health services and perceived social support from health providers, PLHIV, and partners. In mediation analyses, we will estimate both the impact of the intervention on these factors as well as associations between these factors and the post intervention outcomes to partition any observed impacts of treatment into direct and indirect effects. The analytic approach will be based on path analyses using regression analyses or structural equation modeling.

#### Qualitative Analyses

A qualitative data software program, such as QSR Nvivo or QDA Miner, will be used to organize, code, and analyze all qualitative data. Inter-rater reliability checks will be conducted periodically during data coding and analysis, and quantitative coefficients such as Scott’s Pi or Cohen’s Kappa may be used to assess the extent of agreement between coders. Coded data will be analyzed for themes according to the study objectives and research questions. Data will be compared across women, men, and key populations (MSM and FSW) to identify similarities and differences in their reactions to and opinions about the mHealth intervention. Data from facility staff will be analyzed separately from intervention participant data.

#### Cost Effectiveness Analysis

To determine the incremental cost-effectiveness ratio (ICER) of LifeLine, we will relate incremental costs (the numerator) to health effects (the denominator). Specifically, the numerator for the ICER will be calculated as the additional costs associated with implementing the LifeLine program beyond the standard of care. The denominator for the ICER will be calculated from the change in proportion virally suppressed at 12-month follow-up in the LifeLine intervention group compared to the change in proportion virally suppressed in the standard of care group.

Sensitivity analyses will be conducted to model cost-effectiveness under different parameters, such as lower text message costs that may be negotiated with mobile network operators. A tornado diagram will be generated to visually represent the resulting sensitivity analyses to show the costs and savings associated with different scenarios. In addition, the World Health Organization (WHO) threshold for cost-effectiveness [34] of an ICER lower than three times gross domestic product per person will be considered in presenting results from cost-effectiveness analyses. Affordability also will be considered in evaluating cost effectiveness of the intervention; for example, intervention costs and the ICER will be compared to country expenditures on health.

## Results

This study was funded through a competitive call for Implementation Science Research to Support Programs under the President’s Emergency Plan for AIDS Relief (PEPFAR) Round 2. However, leading up to protocol review and approval, continued funding for the mHealth intervention under study became uncertain; as a result, there was not a guarantee that the intervention would remain active for the entire study period. Therefore, the funder and the study implementer made a mutual decision to terminate the study, and the study was terminated prior to ethics approval of the protocol. We are currently seeking funding from alternate sources to implement this study.

## Discussion

### Summary

To our knowledge, the current study would be the first clinical trial to examine the effects of mHealth for HIV prevention and care among PLHIV in a scaled-up public sector setting. Results from this study would inform development of guidelines for achieving improved clinical benefits and recommendations for mHealth service delivery, from the clinic to national and international policy levels. Findings also would contribute to understanding how mobile technology interventions motivate better health, as well as how they function when integrated into larger health systems. Furthermore, this study protocol may provide guidance to HIV and mHealth experts who are seeking to evaluate mobile phone programs for HIV prevention, care, and treatment. The protocol focuses on mHealth impact at the clinical, care, and behavioral levels; investigation of implementation issues unique to mHealth interventions; and the costing component all are priorities for evidence generation in the area of mobile phones for health.

### Limitations

There are a few study limitations worth noting. Supplementing routine data collection for ART clients with study-specific data collection reduces costs and participant burden; however, in collaboration with partners and facilities, great care will need to be taken to ensure high quality data, whether routine or study-specific, and across all time points. Self-reported adherence to ART has notable limitations, and we will complement self-reported data with objective measures from pharmacy, clinician, and laboratory data to provide a triangulated perspective on ART adherence. The health behavior constructs we will assess have rarely been evaluated in mobile phone interventions, but focus group data from LifeLine pilot participants suggests that the proposed constructs are impacted by mHealth programs. Finally, we will not be able to control external factors that may confound study results, such as medication supplies or health system challenges, civil or political influences, or other health or technology programs occurring in study communities during the intervention period, although we will document these contextual factors to help with interpretation of findings.

### Conclusions

This study is poised to make a substantive contribution to the evidence base for using mHealth strategies to improve HIV service delivery. Study results may advance the field of HIV service delivery by providing: appropriate and robust scientific methodologies for evaluating mHealth interventions for care and treatment; guidelines for achieving improved clinical benefits for specific populations (eg, women, key populations, time on ART) from mHealth reminders for care and treatment; recommendations (including cost-effectiveness) for service delivery programs and health systems that are planning or implementing mHealth interventions for care and treatment; and improved understanding of how mobile technology interventions motivate better health through use of behavioral, social, and cognitive strategies.

## Abbreviations

ART: antiretroviral therapy
CD4: cluster of differentiation four
FSW: female sex worker
GEE: generalized estimating equations
GHS: Ghana Health Services
HIV: human immunodeficiency virus
ICC: intra-class correlation coefficient
ICER: incremental cost-effectiveness ratio
ITT: Intervention Tracking Tool
mHealth: mobile phones for health
MSM: men who have sex with men
NACP: National AIDS Control Program
PEPFAR: President’s Emergency Plan for AIDS Relief
PLHIV: people living with HIV
RCT: randomized controlled trial
RNA: ribonucleic acid
SHARPER: Strengthening HIV/AIDS Partnerships with Evidence-based Results
SMS: short message service
STI: sexually transmitted infection
USAID: United States Agency for International Development
WHO: World Health Organization
